# Do Wellness Tourists Get Well? An Observational Study of Multiple Dimensions of Health and Well-Being After a Week-Long Retreat

**DOI:** 10.1089/acm.2016.0268

**Published:** 2017-02-01

**Authors:** Marc M. Cohen, Fiona Elliott, Liza Oates, Adrian Schembri, Nitin Mantri

**Affiliations:** ^1^ School of Health and Biomedical Sciences, RMIT University, Bundoora, Victoria, Australia.; ^2^ Cogstate Limited, Melbourne, Victoria, Australia.; ^3^ School of Science, RMIT University, Bundoora, Victoria, Australia.

**Keywords:** wellness tourism, lifestyle, depression, anxiety, stress, sleep, cognitive function, organic food

## Abstract

***Background:*** Wellness retreats use many complementary and alternative therapies within a holistic residential setting, yet few studies have evaluated the effect of retreat experiences on multiple dimensions of health and well-being, and no published studies have reported health outcomes in wellness tourists.

***Objectives:*** To assess the effect of a week-long wellness-retreat experience in wellness tourists.

***Design:*** A longitudinal observational study with outcomes assessed upon arrival and departure and 6 weeks after the retreat.

***Setting:*** A rural health retreat in Queensland, Australia.

***Interventions:*** A holistic, 1-week, residential, retreat experience that included many educational, therapeutic, and leisure activities and an organic, mostly plant-based diet.

***Outcome measures:*** Multiple outcome measures were performed upon arrival and departure and 6 weeks after the retreat. These included anthropometric measures, urinary pesticide metabolites, a food and health symptom questionnaire, the Five Factor Wellness Inventory, the General Self Efficacy questionnaire, the Pittsburgh Insomnia Rating Scale, the Depression Anxiety Stress Scale, the Profile of Mood States, and the Cogstate cognitive function test battery.

***Results:*** Statistically significant improvements (*p* < 0.05) were seen in almost all measures (*n* = 37) after 1 week and were sustained at 6 weeks (*n* = 17). There were statistically significant improvements (*p* < 0.001) in all anthropometric measures after 1 week, with reductions in abdominal girth (2.7 cm), weight (1.6 kg), and average systolic and diastolic pressure (−16.1 mmHg and −9.3 mmHg, respectively). Statistically significant improvements (*p* < 0.05) were also seen in psychological and health symptom measures. Urinary pesticide metabolites were detected in pooled urine samples before the retreat and were undetectable after the retreat.

***Conclusion:*** Retreat experiences can lead to substantial improvements in multiple dimensions of health and well-being that are maintained for 6 weeks. Further research that includes objective biomarkers and economic measures in different populations is required to determine the mechanisms of these effects and assess the value and relevance of retreat experiences to clinicians and health insurers.

## Introduction

Travel and tourism is one of the world's largest industries, with an estimated US$7.6 trillion generated in 2014 (10% of global gross domestic product) and current growth that is faster than seen in the automotive, financial services, and healthcare sectors.^1^ Wellness tourism is a rapidly growing niche segment that is estimated to have generated $438.6 billion in revenues in 2013, with growth projections that are nearly 50% faster than for overall global tourism.^2^ The growth of wellness tourism is driven by demographic and lifestyle trends that include escalating health costs; recognition of lifestyle as a major cause of chronic disease;^3^ and wealthier, health-conscious, proactive consumers with increasing willingness to embrace alternative and holistic therapies.^2,4^ This growth is paralleled by the growth of the global wellness industry, which in 2013 was estimated to represent a $3.4 trillion industry cluster that includes beauty, antiaging, fitness, nutrition, complementary and alternative medicine, preventive and personalized medicine, wellness-lifestyle real estate, spas, thermal and mineral springs, workplace wellness, and wellness tourism.^5^

## The Illness-Wellness Continuum

While the concept of wellness is still evolving, it is generally recognized that wellness is a holistic concept best represented as a continuum, with sickness, premature death, disability, and reactive approaches to health on one side and high-level wellness, enhanced health, and proactive approaches to health and well-being on the other. It is further acknowledged that wellness is multidimensional and includes physiologic, psychological, social, ecologic, and economic dimensions.^6^ These multiple dimensions make wellness difficult to accurately assess as multiple subjective and objective measures are required to account for the different dimensions. Thus, the assessment of wellness in individuals may include a variety of factors, including assessment of physiologic functioning, anthropometry, happiness, depression, anxiety, mood, sleep, health symptoms, toxic load, neurocognitive function, socioeconomic status, social connectivity, and perceived self-efficacy.

Just as wellness exists along a continuum, wellness tourism facilities encompass a range of offerings: from medical tourism facilities based on curative approaches and medical interventions, to wellness tourism facilities based on preventive approaches, lifestyle, and fostering self-responsibility. The provision of wellness retreats is a unique sector within wellness tourism that has evolved from a convergence of hospitality, leisure, resort, and tourism offerings that have become integrated with residential weight loss, drug and alcohol rehabilitation, and conventional and complementary health services. Wellness retreat facilities generally offer residential experiences that last beyond a weekend getaway and involve an immersive experience that is designed to enable and enhance peoples' physical, psychological, spiritual, and/or social well-being.^4^

It has been suggested that people attending retreats do so for many reasons, including indulgence, escape and relaxation, improved physical health and appearance, transcendence, and re-establishment of their self-esteem.^7^ Retreat experiences may include healthy meals and connection with nature; various educational programs and activities such as yoga, meditation, and *t'ai chi*; services such as massage, saunas, and beauty treatments; various heath modalities and treatments; and activities designed to enhance connection between guests. A survey of retreat operators suggests the retreat industry is relatively unique and place-specific, and while there is no standard formula for retreat offerings, most retreats include common elements and a sense of respite, rest, quiet reflection, rejuvenation, and an opportunity to regain good health.^8^

## Retreat-Based Research

To date, most information on wellness tourism comes from reports of international research organizations, government policy documents, and the media because there has been very little peer-reviewed research into wellness tourism by leisure, tourism, or health science researchers.^4^ Thus, while health and wellness retreats are commonly marketed based on purported improvements in health and well-being, only a few published studies have reported on health outcomes from retreat experiences, most of which have focused on specific measures of cognition, metabolism, or subjective well-being. One recent quasi-randomized controlled trial reported improvements in spirituality, gratitude, self-compassion, and anxiety in women who participated in a 5-day resort-based Ayurvedic, yoga, and meditation program compared with women randomly assigned to attend the same resort facility without participating in the program.^9,10^ These results suggest that retreat participants enjoyed benefits over and above the “vacation effect” from being away from routine domestic and work activities. A further report by the same research group documents changes in phospholipid biosynthesis, choline metabolism, and lipoprotein metabolism pathways in retreat participants, thus providing mechanistic insights into health improvements from retreat experiences.^11^ A further cross-sectional cohort study suggests that retreat experiences may lead to improved cognitive function with improved measures of task-based executive attention observed after an intensive week-long meditation retreat.^12^

In addition to direct evidence of health benefits from retreat experiences, benefits may be inferred from research into the separate components of retreat experiences. Retreats are commonly located in natural environments, and considerable evidence suggests that exposure to natural environments results in enhanced physical, mental, social, and spiritual health.^13–16^ These effects are likely to be mediated through multiple mechanisms that include improved air quality, exercise, social cohesion, and stress reduction^16^ and are further enhanced by health retreat activities with documented health benefits, such as yoga,^17,18^ meditation,^19,20^ mindfulness,^21^ *t'ai chi*,^22,23^ and massage.^24,25^ Health improvements are also likely to result from increased physical activity and healthy eating, which often includes an emphasis on plant-based, organically produced food.

Along with access to healthy food, activities, and environments, retreat experiences also commonly provide educational programs that encourage personal growth and support the adoption of healthy lifestyle practices, as well as providing a variety of treatments and therapeutic modalities scheduled along with activities that are intended to reduce stress and enhance sleep duration and quality. Providing an environment conducive to reducing stress and enhancing sleep quality has many benefits. Poor sleep quality and duration are associated with a high “allostatic load” and have been linked to a range of chronic diseases.^26^ An environment conducive to rest, respite, and relaxation may therefore lead to improvements in multiple health conditions, along with improved mood and cognition. Such improvements are suggested by an Austrian study showing that sleep quality improved during a 3-week resort stay.^27^ Sleep quality was also noted to improve in medical students after a week-long retreat aimed at encouraging a healthy lifestyle.^28^

Although there is little published research on the health benefits of retreat participation among general wellness tourists, observational studies provide limited evidence for benefits in specific populations. For example, a week-long residential retreat incorporating photographic art therapy in concert with psychoanalytically oriented group therapy and mind–body practices resulted in significant and sustained reductions in psychological distress and improvements in spiritual well-being and quality of life in women with breast cancer.^29^ Similarly, a prospective randomized multicenter trial of European patients with nonmetastatic breast cancer treated by chemotherapy found that a 2-week spa intervention led to improved quality of life and other measures that were maintained for more than 6 months.^30^ A U.S. study of patients with multiple sclerosis reported improvement in the mental component of quality of life after a 1-week retreat,^31^ and Australian studies of patients with multiple sclerosis found significant positive effects on health-related quality of life 1, 2.5, and 5 years after a 5-day residential retreat program, despite the usual deterioration seen in this condition.^32,33^

Retreat participation has also improved cardiovascular risk factors and spirituality. A Czech study of 1349 volunteers reported significant improvements in body weight, body–mass index, blood pressure, serum cholesterol, and blood glucose immediately after a 1-week retreat that included a low-fat, low-energy, lacto-ovo-vegetarian diet and exercise.^34^ Similarly, increased spirituality, well-being, meaning in life, and confidence in handling problems in cardiac patients and their partners was reported in a study of a 2.5-day educational lifestyle retreat.^35^

While the above studies suggest that retreat participation may improve a variety of health and wellness measures, there is a lack of studies on the effect of retreat experiences on multiple dimensions of health and well-being. No published studies have reported on the health outcomes of general wellness tourists. To address this gap the current study was performed to assess the health and wellness effect of a week-long wellness retreat experience in wellness tourists.

## Materials and Methods

### Aim

This study aimed to assess any changes in multiple dimensions of health in a group of wellness tourists attending a week-long health retreat.

### Design and setting

This longitudinal, observational study was done among guests participating in a 1-week residential retreat at the Gwinganna Lifestyle Retreat in Queensland, Australia, with measures taken immediately before and after the retreat and 6-weeks after the retreat. The study was approved by the RMIT University Human Research Ethics Committee. Because this study was an observational study and not a clinical trial, it was not registered as such.

### Participants

Participants were recruited from guests attending a regularly scheduled 7-day program at the Gwinganna Lifestyle Retreat in the Tallebudgera Valley in Queensland. All guests who had booked to attend a specific week-long program were invited to participate in the study. Guests were emailed information about the study before arriving at the retreat center and were provided with further information upon arrival. Informed consent was obtained from all participants and confirmed by having participants sign a consent form.

### Intervention

The program consisted of a 7-day residential program where all food, beverages, and a wide range of activities were provided. Many optional activities were provided between 6 am and 2 pm, followed by “dreamtime”—an opportunity for participants to rest, rejuvenate, and receive spa treatments, including massage, body treatments, counseling sessions, and other healing modalities. Morning activities were provided before breakfast from 6:00 am and consisted of “vigorous” or “restorative” activities. The vigorous activities included high-energy physical activities such as challenging nature walks, boxing, dance, and spin classes. Restorative activities included more gentle physical activities, such as *qi gong*, yoga, Pilates, meditation. and gentle nature walks. After breakfast, further activities were offered, and a wellness-related educational talk (on such issues as diet, physical activity, posture, elimination, relationships) was scheduled before lunch. All activities were optional, and participants could choose to attend only activities that suited them or undertake their own activities within the 100-acre property, which offered many nature walks and peaceful places to rest, as well as a large well-equipped gymnasium and several heated outdoor pools. All meals were prepared with organic ingredients, many of which were sourced from the onsite organic garden and were designed to enhance taste and enjoyment by an expert chef. The diet consisted of mainly plant-based foods with some fish and egg protein, with no added sugar or salt, and no gluten, dairy, caffeine, alcohol, red meat, or canned or packaged food.

The week-long experience was structured with a focus on encouraging a change in behavior through regular habit-forming activities and an emphasis on establishing a regular circadian routine and sleep pattern. The retreat also offered many opportunities to foster a connection with nature and enhance social connections with like-minded people while avoiding news, electronic media, and the use of digital technology. The retreat staff also strived to maintain a culture of well-being throughout the experience, and trained health professionals were on hand to address any health issues that arose.

### Outcome measures

Participants recorded their demographic details (age, sex, postal code, and previous retreat experiences) and had anthropometric measures (height, weight, abdominal girth, and blood pressure) and a urine sample taken upon arrival and before departure. Urine samples were immediately frozen and transported to RMIT University, where they were kept at −80°C. Samples were thawed and pooled to created two pooled samples: one pool containing urine obtained on arrival and a second pool containing urine obtained upon departure. The pooled samples were refrozen before being sent to AsureQuality Laboratories in New Zealand for analysis of dialkylphosphate (DAP) metabolites using gas chromatography tandem mass spectrometry, and creatinine correction was applied according to a method that has been previously described.^36^ Participants also completed several questionnaires and a cognitive function test on arrival, before their departure, and 6 weeks after their retreat stay (Table 1).

**Table 1.** Timing and Frequency of Assessment Measures

*Assessment measures*	*Day 1 of retreat*	*Day 7 of retreat*	*6 wk after retreat*
Anthropometric measurements	X	X	
Urinary pesticide residues	X	X	
Five Factor Wellness Inventory	X	X	X
Depression, Anxiety Stress Scales	X	X	X
Generalized Self-Efficacy Scale	X	X	X
Health symptoms	X	X	X
Dietary behavior	X	X	X
Cogstate cognitive function test battery	X	X	X
Pittsburgh Insomnia Rating Scale	X	X	X
Profile of Mood States	X	X	X

Several questionnaires were used to assess general health, dietary habits, depression, anxiety, stress, mood, sleep quality, and self-efficacy. General health and well-being measures included the Five Factor Wellness Inventory (FFWI), which is an extensive 98-item questionnaire covering areas of well-being that are grouped under five factors (the creative self, the coping self, the social self, the physical self, and the essential self).^37^ A basic food questionnaire examined dietary behaviors, including participants' overall dietary approach (e.g., vegan, vegetarian, omnivore), along with the extent of organic, packaged food and animal proteins consumed in the previous 3–4 days.

The General Self Efficacy 10-item self–rated questionnaire assessed the participant's perceived ability to cope in difficult situations.^38^ Health symptoms were recorded by using a 41-item, self-rated questionnaire that assessed the frequency and severity of health symptoms relating to the head, eyes, ears, nose, mouth/throat, skin, heart/lungs, digestive tract, joints/muscles, weight, energy, mind/emotions, and other. Sleep quality was measured by using the Pittsburgh Insomnia Rating Scale (PIRS_20), which is a 20-item self-reported questionnaire assessing sleep quality and insomnia over a 7-day period.^39^ The Depression Anxiety Stress Scale (DASS) was used to assess stress, anxiety, and depressive symptoms,^40^ and the Profile of Mood States (POMS) was used to assess mood over the previous 24 hours.^41^

Cognitive function was assessed by using the Cogstate Brief Battery, which is a brief battery of four computerized tasks that measure various aspects of cognition (www.cogstate.com). This battery typically requires 12–15 minutes to complete and contains four tasks that measure psychomotor function (Detection), attention (Identification), visual learning (One Card Learning), and working memory (One Back). Each of the tests has a similar design and uses playing cards (e.g., ace, queen, king) as visual stimuli and requires only a “yes” or “no” response from the participant using the keyboard. The Detection task is a simple reaction time that asks the participant to press “yes” as soon as the card flips over. The Identification task is an attention task that requires a “yes” response if the card is red and a “no” response if the card is black. One Card Learning is a visual learning task that asks participants to press “yes” if they have seen the current card in this test, or “no” if they have not seen the current card in this test. One Back is a working memory task that requires a “yes” response if the current card is the same as the previous card, and a “no” response if the current card is different to the previous card. Performance on the Detection, Identification and One Back tasks is measured by using reaction time, and performance on One Card Learning is measured by using accuracy.

### Statistical analysis

The Statistical Package for the Social Sciences (SPSS), version 22 (IBM, Armonk, NY), was used for all statistical analyses. Descriptive statistics were used to review the demographic characteristics of the sample, anthropometric measurements, scores on the psychological and health symptom scales, and performance on the cognitive battery. Change in performance over the 7-day intervention and 6-week follow-up were evaluated by using paired-samples *t*-tests for the anthropometric measures, psychological and health symptom scales, and metabolite outcomes. Performance on the cognitive battery was analyzed by computing the proportion of the sample with a subtle cognitive impairment at each assessment, with a subtle impairment classified as a z-score less than −0.5 relative to each participant's same-age peers. To evaluate whether participants with better or poorer health at baseline responded differently to the intervention, the sample was clustered into two subgroups (<25th percentile, >75th percentile at baseline) on each anthropometric, psychological, health, and cognitive measure, with independent-sample tests used to analyze differences in change scores between day 1 and day 7 across these two groups.

## Results

Of the 47 guests that attended the retreat during the study week, 37 agreed to participate in the study and provided anthropometric data. Of these, 32 participants provided questionnaire data. Seventeen of these participants completed the follow-up cognitive function testing and 16 the follow-up questionnaires 6 weeks after the retreat. The baseline demographic details of participants are provided in Table 2. The average age of participants was 48.7 years, with the majority (65%) being female. Most participants had university-level education (64.5%), were omnivorous (94.5%), and had consumed less than 25% of organic food in the previous 3–4 days (89%).

**Table 2.** Demographics of Participants

*Characteristic*	*Value*
Mean age (SD) (yr)	48.70 (14.65)
Men	13 (35)
Attended Gwinganna or similar retreat before?	
No	16 (43)
Yes, more than 12 mo ago	15 (40.5)
Yes, once in past 12 mo	5 (13.5)
Yes, more than once in past 12 mo	1 (3)
Highest level of education	
Less than high school	1 (3)
High school graduate	8 (21.5)
Trade/technical school/associate's degree	4 (11)
Bachelor's degree	16 (43)
Advanced degree	8 (21.5)
Which of the following best describes your diet?	
Vegan: plant-based foods only	0
Vegetarian: primarily plant-based foods but some dairy/eggs	2 (5.5)
Omnivore: assortment of meat, seafood, vegetables, dairy, grains	29 (78)
Carnivore: meat, seafood and dairy several times a week	4 (11)
Top of the food chain: meat, seafood or dairy at almost every meal	2 (5.5)
In the past 3–4 days, what percentage of your diet was organic food?	
<5%	21 (57)
5%–25%	12 (32)
25%–75%	3 (8)
>75%	1 (3)

Unless otherwise noted, values are *n* (%).

SD, standard deviation.

Anthropometric data are presented in Table 3. There were statistically significant (*p* < 0.001) improvements in all anthropometric measures after the 7-day retreat. Average abdominal girth was reduced by 2.7 cm (from an average of 90.7 cm [standard deviation (SD), 13.5] to an average of 88.1 cm [SD, 12.6]), average weight was reduced by 1.6 kg (from an average of 80.4 kg [SD, 16.7] to an average of 78.8 kg [SD, 15.5]), average systolic blood pressure was reduced by 16.1 mmHg (from an average of 142.2 mmHg [SD, 16.5] to an average of 126.1 mmHg [SD, 13.8]), and average diastolic blood pressure was reduced by 9.3 mmHg (from an average of 83.8 mmHg [SD, 8.4] to an average of 74.4 mmHg [SD, 10.2]).

**Table 3.** Summary of Anthropometric Measurements at Baseline and After Retreat

	*Day 1 (*n* = 37)*			*Day 7 (*n* = 37)*			
*Variable*	*Mean (SD)*	*25th percentile*	*75th percentile*	*Mean (SD)*	*25th percentile*	*75th percentile*	p-*Value*
Girth (cm)	90.7 (13.5)	80.5	99.8	88.1 (12.6)	79.5	95.6	<0.001
Weight (kg)	80.4 (16.7)	64.9	91.6	78.8 (15.5)	64.2	89.0	<0.001
Systolic blood pressure (mmHg)	142.2 (16.5)	132.0	153.5	126.1 (13.8)	118.0	132.5	<0.001
Diastolic blood pressure (mmHg)	83.8 (8.4)	78.5	88.0	74.4 (10.2)	66.0	83.0	<0.001

The questionnaire scores are presented in Table 4. All the questionnaire measures with the exception of health symptom frequency and severity showed a statistically significant improvement after the 7-day retreat. The more limited 6-week follow-up data suggest that health symptom frequency and severity continued to improve and became statistically significant, while the differences in the DASS and PIRS reduced somewhat and were no longer statistically significant after 6 weeks, even though they remained below pre-retreat levels.

**Table 4.** Summary of Questionnaire Results

*Measure*	*Day 1 of retreat (*n* = 32)*	*Day 7 of retreat (*n* = 32)*	*6 wk after retreat (*n* = 16)*
FFWI	181.2 (30.6)	166.0 (30.6)^**^	152.13 (31.20)^**^
PIRS	40.7 (13.3)	33.2 (9.8)^**^	34.19 (12.77)
DASS	63.8 (24.6)	48.5 (10.9)^**^	51.06 (17.37)
POMS	78.3 (24.8)	58.2 (12.5)^**^	61.81 (21.97)^*^
HSQ (frequency)	59.0 (12.6)	54.8 (8.0)	53.44 (8.69)^*^
HSQ (severity)	21.9 (14.3)	16.2 (13.5)	12.94 (8.75)^**^
GSE	32.15 (4.54)	33.48 (4.04)^*^	35.19 (3.47)^*^

Values are mean (SD).

^*^ *p* < 0.05 for paired-samples *t*-test with day 1 of retreat.

^**^ *p* < 0.01 for paired-samples *t*-test with day 1 of retreat.

FFWI, Five Factor Wellness Inventory; PIRS, Pittsburgh Insomnia Rating Scale; DASS, Depression, Anxiety Stress Scales; POMS, Profile of Mood States; GSE, Generalized Self-Efficacy Scale; HSQ, Health Symptom Questionnaire.

The results of Cogstate cognitive function testing are presented in Table 5. The Cogstate results measuring speed and accuracy showed a nonsignificant improvement after the retreat. The first three indicators (Detection, Identification, and One Back) relate to reaction time; all three showed a fractional decrease from day 1 to day 7. The fourth indicator (One Card Learning) relates to accuracy and showed a fractional increase from day 1 to day 7. The *p*-values showed no significant changes, at 0.38, 0.79, 0.08, and 0.14, respectively. Cognitive impairment was evaluated by comparing performance of the current sample with normative data. Impairment was defined as a z-score of less than −0.5 on the cognitive tasks. Table 6 shows performance for each task in the Cogstate battery individually, as well as the proportion impaired on two or more of the four tasks in the battery. This indicates that 60.61% of participants had a −0.5 level impairment on two or more tasks at baseline, whereas only 37.5% were impaired at this level after the week-long retreat.

**Table 5.** Number of Participants with Mild Cognitive Impairment at Baseline, After Retreat, and 6-Week Follow-Up on Each of the Four Cognitive Assessment Measures

*Cognitive task*	*Day 1 of retreat (*n* = 33)*	*Day 7 of retreat (*n* = 32)*	*6 wk after retreat (*n* = 17)*
Detection	11 (33.33)	10 (31.25)	8 (47.06)
Identification	13 (39.39)	9 (28.125)	6 (35.29)
One Back	18 (54.55)	15 (46.875)	8 (47.06)
One Card Learning	16 (48.48)	11 (34.375)	5 (29.41)
Two or more tasks impaired^a^	20 (60.61)	12 (37.5)	9 (52.94)

Values are *n* (%). Cognitive impairment was defined as a z-score of 0.5 SDs below the aged-matched normative mean, indicative of subtle impairment relative to the participant's same-age peers.

^a^ Performance was <0.5 SDs below the age-matched normative mean on two or more cognitive tasks.

**Table 6.** Summary of Change Scores for the Lowest and Highest Quartile for Psychological, Anthropometric, and Cognitive Outcomes

*Measure*	*Lowest quartile^a,b^*	*Highest quartile^b,c^*	t*-Test*	*Cohen's* d
Weight	0.00 (0.93)	−3.14 (2.05)	4.20^*^	1.97
Systolic BP	−5.00 (11.15)	−27.55 (12.89)	4.27^**^	1.86
Diastolic BP	−5.33 (8.25)	−12.18 (9.38)	1.71	0.77
DASS	−0.38 (8.33)	−41.5 (21.02)	5.15^**^	2.57
PIRS	−1.22 (7.34)	−19.5 (13.11)	3.60^*^	2.94
FFWI	−12.75 (25.12)	−24.50 (29.22)	0.86	0.43
POMS	−11.13 (5.46)	−46.25 (22.96)	4.21^**^	2.10
Health frequency	4.50 (17.54)	−13.38 (14.68)	2.21^*^	1.11
Health severity	10.38 (25.74)	−22.63 (16.66)	3.04^**^	1.52
Cogstate battery				
Detection	0.05 (0.05)	−0.03 (0.04)	3.24^*^	1.80
Identification	0.07 (0.07)	−0.05 (0.06)	3.68^*^	1.85
One Card Learning	−0.00 (0.05)	−0.11 (0.07)	3.31^*^	1.79
One Back	0.01 (0.05)	−0.02 (0.05)	0.97	0.60

^a^ Participants in the first to 25th percentiles at day 1 (better health outcomes at day 1).

^b^ The mean represents the mean change between day 1 and day 7.

^c^ Participants in the 75th to 99th percentiles at day 1 (poorer health outcomes at day 1).

^*^ *p* < 0.01.

^**^ *p* < 0.001.

BP, blood pressure.

Table 6 presents the change scores between day 1 and 7 for the lowest and highest quartiles for the anthropometric, psychological, health symptom, and cognitive outcomes. These data show that compared with participants in the lowest quartile at baseline (highest function), those in the highest quartile at baseline (poorest function) demonstrated significant improvements on all measures except the FFWI, diastolic blood pressure, and One Back cognitive test, with the FFWI and diastolic blood pressure improving in both groups. For most measures, the change scores indicate a large effect, with changes being greater than 1 SD in most measures and greater than 2 SDs for the DASS, PIRS, and POMS as indicated by the Cohen's d effect sizes.

Table 7 presents the data on DAP metabolites in the pooled urine samples. These demonstrated that all metabolites could be detected before the retreat and were either not detectable or not quantifiable after the retreat.

**Table 7.** Creatinine-Corrected Results for Urinary Dialkylphosphate Metabolites

*Analyte*	*LOD (μg/L)*	*LOQ (μg/L)*	*On arrival (μg/g creatinine)*	*On departure (μg/g creatinine)*
Dimethylphosphate	0.42	1.3	1.9	NQ
Diethylphosphate	0.51	1.5	NQ	NQ
Dimethylthiophosphate	0.28	0.83	2.0	ND
Diethylthiophosphate	0.16	0.49	1.1	ND
Dimethyldithiophosphate	0.14	0.43	NQ	ND
Diethyldithiophosphate	0.11	0.33	NQ	ND

Creatinine analysis performed by Aotea Pathology, New Zealand.

LOD, limit of detection; LOQ, limit of quantification; NQ, not quantified, levels greater than or equal to the LOD and less than the LOQ; ND, not detected, levels below the LOD.

## Discussion

This is the first study to report on a wide range of multidimensional wellness measures such as anthropometrics, depression, anxiety, stress, mood, sleep quality, health symptoms, cognitive function, and urinary pesticide metabolites in wellness tourists attending a health retreat. The finding of statistically significant and clinically relevant improvements, with greater improvement in the most unhealthy participants upon arrival, suggests that an integrated lifestyle experience was effective in improving multiple dimensions of health and well-being. Furthermore, the finding of no detectable levels of organophosphate pesticide metabolites in pooled urine samples after the retreat suggests that the retreat experience fostered the detoxification and elimination of pesticide residues, while avoiding further exposure. This finding is consistent with previous studies on organic diets.^36^

It is interesting to note that while all measures improved after the retreat, the improvement of various measures over time differed. The Five Factor Wellness Inventory, which was the most comprehensive and multidimensional measure, displayed the greatest improvement, with statistically significant improvements (at *p* < 0.01) at both 7 days and 6 weeks. Similarly, mood and self-efficacy displayed significant improvements that were sustained at 6 weeks. In contrast, improvements in health symptom severity and frequency were not statistically significant after 7 days, yet became significant after 6 weeks; the significant improvements seen in the PIRS and the DASS after 7 days were not sustained at 6 weeks despite remaining below pre-retreat levels.

It is likely that many factors contributed to these results, including the effects of specific activities and the general effects of enjoyment and relaxation. Enjoyment is associated with better psychological and physiological well-being,^42^ and the retreat experience was set in a beautiful natural environment and included educational, therapeutic and leisure activities that were specifically designed to be enjoyable. The retreat also provided the opportunity for relaxation, reflection, and recovery by providing a break from participants' usual routine. Limiting the use of electronic media and devices, as well as supporting natural circadian rhythms by having participants rise shortly after dawn and retire shortly after nightfall, probably contributed to the improvements in perceived stress and sleep immediately after the retreat, with improved sleep contributing to improvements in cognition. Because it is unlikely that participants were able to maintain all of these measures once they returned home, it is perhaps not surprising that the PIRS and DASS scores drifted back toward pre-retreat levels once participants returned to the routine pressures of life.

Together these findings suggest that the retreat experience helped participants gain control over their life and make positive adjustments to their lifestyles that led to health improvements that continued after they returned to their regular routines. Furthermore, the finding that severity of health symptoms improved more than their frequency suggests that while participants continued to experience health symptoms after the retreat, albeit at a reduced rate, either the actual symptoms diminished, or participants were better able to cope, leading to a perceived reduction in severity. The improvement in the frequency and severity of existing health symptoms and other outcomes after 6 weeks further suggests that the retreat experience helped prevent or diminish the impact of existing health complaints and any associated lifestyle-related chronic diseases.

The ability of a residential retreat experience to address chronic disease was explored by a longitudinal study of nearly 24,000 individuals who participated in the German Socio-Economic Panel Study and attended up to 3 weeks of residential “spa treatment” provided by German statutory health insurance. After controlling for individual fixed effects, this study found significant benefits from residential spa treatments in terms of lost workdays and the number of days in a hospital, leading the authors to suggest that public and private insurers may benefit from providing access to such services.^43^ While Germany and some parts of Europe include retreat-type experiences within their health system, retreat facilities in most countries sit outside the mainstream health system and retreat participation is not supported by insurance cover or tax incentives. Thus, wellness industry stakeholders around the world have expressed concern that health insurers do not recognize the importance of wellness retreats or the complementary and alternative therapies that they offer, despite the need for lifestyle-based interventions to quell the tide of chronic disease.^4^ Further research is therefore needed to determine the economic value of providing health insurance coverage and/or tax incentives for retreat participation, as well as to establish the effectiveness of retreat experiences in addressing different medical conditions.

The results of this study suggest that a week-long wellness retreat offers multiple health benefits, yet the study has many limitations. While the study included psychological, anthropometric, cognitive, and clinical measures, it did not record anthropometric measures at 6 weeks or measure economic outcomes, specific disease biomarkers, or underlying molecular mechanisms. Furthermore, the study population was drawn from a self–selected group with the time and disposable income to attend a luxury health retreat, and the results may not apply to the wider population. The retreat also offered a wide range of healthy activities, with retreat participants selecting from a range of offerings that were integrated into a seamless week-long experience. It is therefore not possible to determine the influence of any particular activity on producing the observed effects. It is also likely that the effect of each activity differed for individual participants with synergistic interactions between activities that further enhanced their effects.

Because this was an observational study with no comparator group, it is also not possible to distinguish how much of the observed effects were due simply to a break from routine work and domestic activities. The study sample size was also relatively small, yet despite the small sample, the statistically significant improvements observed in multiple health measures suggest effect sizes were large. The 6-week data are further limited by attrition, with only half of the initial participants contributing 6-week follow-up data. Thus, it is not clear whether the participants who provided follow-up data differed significantly from those who did not.

The study is further limited by the number and choice of outcome measures, with the large number of measures used increasing the likelihood of significant differences occurring by chance. Given the number of statistical tests conducted, an adjustment due to multiple testing such as a Bonferroni adjustment was considered; however, given that the *p*-values were highly significant (e.g., <0.01 or <0.001), an adjustment would not have changed the interpretation of results and was therefore deemed unnecessary.

It will be important for future studies to have longer-term follow-up and include economic measures so that the value of retreat experiences can be determined for both individuals and third-party payers, such as governments and health insurers. It will also be interesting for future studies to include measures of specific disease biomarkers and underlying molecular mechanisms. This could be done through the use of next-generation sequencing technologies for comprehensive RNA analysis to document changes in mRNA expression,^44^ microRNA expression,^45^ or long noncoding RNA expression.^46^ Such research would thus build on previous studies that have shown enhanced metabolic activity, including greater mitochondrial resilience^47^ and telomerase activity,^48^ with various lifestyle practices, such as yoga and meditation.^49^ Future studies could also assess DNA polymorphisms for toxin metabolizing genes^50^ and DNA repair genes^51^ within the participants to determine how these polymorphisms affect their ability to revitalize their metabolic and cognitive states. Another genetic aspect of interest is epigenetic changes that lead to altered gene expression. Epigenetic factors, including histone modifications and DNA methylation of genes and their promoters, were recently shown to be governed by lifestyle and environment.^52^ Retreat experiences may therefore act on multiple genes to reverse harmful epigenetic changes caused by stress and toxic insults that people are exposed to in their normal life.^53^

The importance of healthy lifestyles in preventing and treating chronic disease is undisputed. However, while previous studies have focused on specific therapeutic interventions or individual lifestyle practices, retreat environments provide a unique living laboratory where all aspects of lifestyle can be controlled and studied. Retreat experiences provide a unique opportunity for people to escape from unhealthy routines and engage in healthy practices and activities that lead to immediate and sustained health benefits. Research into these experiences may therefore help determine how lifestyle interventions can address the burden of lifestyle-related chronic disease.

## Conclusion

A 1-week residential retreat experience can lead to substantial improvements in multiple dimensions of health and well-being, with many improvements being maintained at 6 weeks. Further, more rigorous research with longer follow-up and larger populations is now needed to confirm these results, and future research that includes objective biomarkers and economic measures is needed to determine the underlying mechanisms, value, and longevity of the observed effects and how they apply to different populations and health conditions.
